# Prognostic value of D-dimer/fibrinogen ratio on in-hospital outcomes of patients with heart failure and COVID-19

**DOI:** 10.2217/bmm-2021-0341

**Published:** 2021-10-20

**Authors:** Selda Murat, Bektas Murat, Muhammet Dural, Gurbet Ozge Mert, Yuksel Cavusoglu

**Affiliations:** ^1^Medical Faculty Department of Cardiology, Eskisehir Osmangazi University, Eskisehir, 26040, Turkey; ^2^Department of Cardiology, Eskisehir City Hospital, Eskisehir, 26080, Turkey

**Keywords:** coronavirus disease, D-dimer/fibrinogen ratio, heart failure

## Abstract

**Aim:** In the present study, the relationship between D-dimer/fibrinogen ratio (DFR) and in-hospital outcomes was evaluated in patients with COVID-19 and a diagnosis of heart failure (HF). **Materials & methods:** In-hospital outcomes were compared in patients with high and low DFR values. **Results:** With regard to in-hospital outcomes, patients in the third tertile of DFR had a higher rate of mechanical ventilation, cardiogenic shock and death (p < 0.001). The length of ICU stay was longer in the third tertile group (p < 0.001). When evaluated together with infection markers, DFR was found to be an independent predictor of outcomes. **Conclusion:** DFR can be used as a prognostic marker in patients with COVID-19 with a diagnosis of HF, and perhaps more valuable than other infection markers.

COVID-19, caused by SARS-CoV-2, is a rapidly spreading pandemic associated with high morbidity and mortality worldwide, with the number of new cases and deaths continuing to rise [1,2]. Although COVID-19 is transmitted primarily through respiratory droplets/aerosols, initially causing pneumonia in the lung, it may affect multiple organs, including the cardiovascular system [3–6]. Previous studies have shown that comorbidities such as hypertension (HT), diabetis mellitus (DM) and cardiovascular disease including heart failure (HF) are associated with poor prognosis [1,7,8]. Some recent studies have shown the role of HF both as a risk factor for a poor clinical course and for increased mortality and as a possible consequence of COVID-19-related myocardial damage [6,9,10]. COVID-19 has been described as a thrombo-inflammatory syndrome [11,12]. Among severely ill patients with mortality, diffuse endothelial dysfunction, widespread coagulopathy and complement-induced thrombosis have resulted in the development of thromboembolism [13]. Several studies show that fibrinogen, D-dimer and fibrinogen degradation products are associated with mortality through coagulation impairment and inflammation in patients with COVID-19. Although some previous studies reported that D-dimer elevation is associated with the severity of COVID-19 [1,14,15], a recent study conducted by Emmanuel *et al.* reported confusion and potential for misinformation regarding D-dimer [16]. Fibrinogen is also well studied in patients with COVID-19. Although it has been shown in some previous studies that fibrinogen is associated with the severity of COVID-19 disease, other studies have reported that fibrinogen alone is not significant and should be evaluated together with D-dimer [17–19]. Some studies have evaluated the value of plasma D-dimer/fibrinogen ratio (DFR) in the diagnosis of pulmonary thromboembolism and lower extremity venous thrombosis [20–22]. DFR also shows a unique value in determining the pathophysiological mechanism of stroke [23] and can predict the prognosis of gastrointestinal stromal tumors in patients hospitalized with HF [24]; however, the prognostic values of DFR in patients with COVID-19 with a history of HF is unclear. Therefore, the present study was designed to evaluate the relationship between DFR and in-hospital prognosis for patients with COVID-19 with a history of HF.

## Material & methods

### Study subjects & design

The study enrolled 240 consecutive patients admitted to Eskisehir Osmangazi University, Medical Faculty Department of Cardiology and Eskisehir City Hospital Department of Cardiology with laboratory-confirmed SARS-CoV-2 infection from 15 March 2020 to 1 December 2020. The diagnosis of COVID-19 was confirmed by RNA reverse-transcriptase polymerase chain reaction (RT-PCR) detection of the SARS-CoV-2 in the clinical laboratory of both centers. The exclusion criteria were age under 18 years old, outpatients who were referred to another hospital during their hospitalization, acute myocardial infarction, patients with a history of cerebral infarct (n = 5), malignancy (n = 2) and patients with acute deep venous thrombosis and/or pulmonary thromboembolism (n = 1). Heart failure was identified from the International Classification of Diseases, tenth revision, clinical modification (ICD-10-CM) codes in patient electronic medical records (EMR), including I50 (heart failure), I50.1 (left ventricular failure, unspecified), I50.2 (systolic [congestive] heart failure) and I50.9 (heart failure, unspecified) diagnostic codes. A total of 232 patients with HF were included in the study.

### Data collection

Demographic characteristics (age and sex); cardiovascular history and chronic diseases including heart failure, arterial hypertension, diabetes mellitus, ischemic heart disease, atrial fibrillation, chronic obstructive pulmonary disease (COPD), chronic kidney disease (CKD), devices (pacemaker and implantable cardioverter-defibrillator); clinical data (vital signs, laboratory findings) and therapy were collected from EMRs. Heart rate, oxygen saturation and blood pressure were recorded at the time of admission. The length of stay in the ICU and ward was obtained from discharge records. Data on counts or levels of hemoglobin (Hb), white blood cells (WBCs), lymphocytes (L), neutrophils (N), sodium, potassium, glucose, alanine transaminase (ALT), aspartate aminotransferase (AST), serum albumin (ALB), serum creatinine (sCr), blood urea nitrogen (BUN) and the highest values of inflammatory markers such as C-reactive protein (CRP), ferritin, lactate and procalcitonin were included. From the D-dimer and fibrinogen values, two values were taken into consideration: The first within 24 hours of admission to hospital and the highest values. D-dimer levels (normal range 0–0.50 mg/l) were measured by immunoturbidimetry and fibrinogen levels (normal range 170–420 mg/dl) by clotting method using the Sysmex CS5100 automated coagulation analyzer. DFR was calculated using the following formula:

$$\text{DFR}=\frac{\text{D}-\text{dimer}\;\left(\mu \text{g}/\text{ml}\right)}{\text{Fibrinogen}\;\left(\text{mg/dl}\right)}\times 100$$

In-hospital mortality, respiratory failure requiring noninvasive mechanical ventilation and orotracheal intubation, duration of ICU stay, acute kidney injury treated with renal replacement therapy, need for blood transfusion and use of intravenous vasopressor drug were evaluated. The clinical outcomes were defined as all-cause death, respiratory failure requiring mechanical ventilation and cardiogenic shock during hospitalization. Cardiogenic shock was defined as hypotension (SBP <90 mmHg) with evidence of hypoperfusion and end-organ dysfunction [25]. The left ventricular ejection fraction (LVEF) before or during index COVID-19 admission was recorded, if available. All data were checked by two researchers to ascertain accuracy.

### Statistical analysis

Normally distributed continuous variables are reported as mean ± standard deviation (SD), while skewed variables are expressed as medians and interquartile ranges (IQRs). Shapiro-Wilk tests were performed and density maps were drawn to determine the normality of the distribution of the continuous variables. Differences between groups were compared by Chi-square test or Fisher's exact test. Comparisons of differences between groups were made by analysis of variance (one-way ANOVA). The Kruskal–Wallis H test was used to compare the groups that did not conform to the normal distribution.

Patients with a history of HF with COVID-19 were grouped into tertiles 1–3. The first tertile included DFR values <0.37, the second tertile included DFR values ranging 0.38–1–1.13 and the third tertile included values higher than 1.13. Patients were also stratified according to receiver operating characteristic (ROC) curve analysis. The optimal cut-off value for serum D-dimer/fibrinogen ratio was calculated as 0.61; therefore, a Kaplan-Meier survival curve was constructed according to the cut-off value for DFR. The log-rank test was conducted to compare differences. The relationship between each variable and the composite of mechanical ventilation, death and cardiogenic shock events was analyzed by Cox proportional hazard regression. All variables that were analyzed in the univariate model were included in the multivariate model to evaluate the comprehensive effects of DFR on the end point event. The hazard ratio (HR) and its 95% CI were calculated. All comparisons were two-tailed, with p < 0.05 considered statistically significant. IBM SPSS Statistics 21.0 (IBM Corp. Released 2012. IBM SPSS Statistics for Windows, Version 21.0. Armonk, NY: IBM Corp.) was used for the analyses.

## Results

In this two-center retrospective study, after exclusion and inclusion criteria, 232 patients with a history of HF with laboratory-confirmed COVID-19 were included in the final analysis. In ROC curve analysis, DFR >0.61 predicted poor outcomes in patients with HF and COVID-19 with a sensitivity of 47.6% and specificity of 90.7%. Patients were categorized in DFR tertiles, first (<0.37), second (0.38–1–1.13) and third (>1.13). The mean age of the study population was 73.2 ± 10 years, with males predominant (158; 68.1%). The presence of DM, HT, CAD, prior history of AF and COPD was similar among both groups. With regard to in-hospital outcomes, patients in the third tertile had a higher rate of requiring mechanical ventilation, cardiogenic shock and death (p < 0.001; Table 1).

**Table 1.** Baseline characteristics, medications, vital signs and laboratory data in patients with COVID-19 and heart failure.

Variables	Total (n = 232)	Tertile 1 (<0.37) n = 79	Tertile 2 (0.38–1.13) n = 76	Tertile 3 (>1.13) n = 77	p-value
Age, years, mean ± SD	73.3 ± 10.1	72.9 ± 9.7	74.8 ± 10.4	73.3 ± 10.1	0.297
Male sex, n (%)	158 (68.1)	54 (68.4)	51 (67.1)	53 (68.8)	0.275
Hypertension, n (%)	198 (85.3)	68 (86.1)	62 (81.6)	68 (88.3)	0.529
Diabetes, n (%)	112 (48.3)	35 (44.3)	35 (46.1)	42 (54.5)	0.341
CAD, n (%)	196 (84.5)	66 (83.5)	65 (85.5)	65 (84.4)	0.561
Atrial fibrillation/flutter, n (%)	78 (33.6)	30 (38)	24 (31.6)	24 (31.2)	0.174
COPD, n (%)	63 (27.2)	26 (32.9)	19 (25)	18 (23.4)	0.171
LVEF, %, ±SD	36.71 ± 8.45	38.5 ± 7.85	35.8 ± 8.62	35.7 ± 8.72	0.115
CKD, n (%)	52 (22.4)	16 (20.3)	16 (21.1)	20 (26.0)	0.037
**Vital signs at hospital admission**, **mean ± SD**					
SBP (mmHg)	112.5 ± 22.0	116.2 ± 23.2	114.0 ± 24.1	107.7 ± 18.6	0.021
Heart rate (bpm)	96.6 ± 20.7	92.3 ± 20.2	97.1 ± 21.7	100.6 ± 19.7	0.023
First oxygen saturation (%)	86.3 ± 8.34	88.2 ± 6.97	86.6 ± 9.22	83.9 ± 8.27	0.005
**Laboratory data, mean ± SD**					
Haemoglobin (g/dl)	12.2 ± 2.27	12.3 ± 2.30	12.7 ± 2.34	11.8 ± 2.11	0.039
WBC (10∧^3^/μl)	11.0 ± 5.31	9.83 ± 4.50	10.6 ± 4.73	12.8 ± 6.18	0.004
Platelet (10∧^3^/μl)	214.7 ± 100.9	224.6 ± 100.5	206.3 ± 75.4	212.7 ± 121.6	0.223
Neutrophil (10∧^3^/μl)	8.8 ± 5.10	8.00 ± 4.52	8.71 ± 4.44	9.95 ± 6.12	0.078
Lymphocyte (10∧^3^/μl)	0.95 ± 0.84	1.04 ± 0.79	1.04 ± 1.04	0.75 ± 0.64	0.001
Glucose (mg/dl)	174.6 ± 78.01	156.5 ± 68.0	163.3 ± 65.0	204.3 ± 90.6	<0.001
eGFR (ml/min/1.73 m^2^)	46.0 (27.0–73.0)	52.0 (29.0–75.0)	50.0 (28.0–71.0)	39.0 (20.0–66.5)	0.148
Sodium (mmol/l)	136.2 ± 6.60	136.1 ± 5.7	136.0 ± 5.8	136.3 ± 8.0	0.599
Potassium (mmol/l)	4.52 ± 0.74	4.55 ± 0.64	4.54 ± 0.83	4.46 ± 0.76	0.608
Total protein (g/dl)	6.25 ± 0.93	6.57 ± 0.94	6.27 ± 0.84	5.89 ± 0.87	<0.001
Albumin (g/dl)	3.4 (3.1–3.90)	3.7 (3.2–4.0)	3.5 (3.1–3.9)	3.2 (3.0–3.6)	<0.001
First fibrinogen (mg/dl)	467.8 ± 178.7	454.4 ± 178.0	500.4 ± 177.4	449.3 ± 178.5	0.261
First procalsitonin (ng/ml)	0.26 (0.1–2.07)	0.15 (0.05–0.28)	0.29 (0.11–3.30)	0.61 (0.20–5.07)	<0.001
First D-dimer (ng/ml)	1.1 (0.56–3.6)	0.71 (0.43–1.11)	1.53 (0.61–3.26)	3.67 (0.72–8.60)	<0.001
First ferritin (ng/dl)	287.0 (114.5–682.0)	173.9 (78.5–505.8)	295.0 (104.0–678.0)	423.4 (196.4–842.0)	<0.001
First LDH (u/l)	288.5 (214.0–474.5)	254.0 (205.0–365.0)	296.0 (212.0–491.0)	366.0 (251.2–567.5)	<0.001
First CRP (mg/l)	62.6 (16.32–133.0)	36.9 (11.1–86.0)	74.3 (16.5–150.7)	88.5 (35.2–158.4)	<0.001
Highest CRP (mg/l)	117.15 (58.25–190.75)	83.1 (40.0–151.9)	132.9 (77.4–191.9)	146.0 (82.4–222.0)	0.001
Highest troponin (ng/l)	135.9 (31.6–383.3)	68.0 (14.7–270.1)	157.5 (44.2–393.5)	177.2 (59.0–487.3)	0.002
Highest ferritin (ng/dl)	628.0 (362.25–1474.2)	448.5 (254.7–769.0)	625.0 (386.0–1501.0)	1002.0 (481.0–2329)	<0.001
Highest D-dimer (ng/ml)	3.2 (1.17–7.99)	0.89 (0.66–1.61)	3.21 (2.61–4.65)	9.14 (6.86–16.27)	<0.001
Highest fibrinogen (mg/dl)	529.5 ± 219.4	530.0 ± 223.1	537.4 ± 198.7	521.1 ± 237.6	0.809
Highest LDH, (u/l)	450.0 (293.0–632.5)	356.0 (240.5–491.0)	430.0 (346.0–602.0)	575.0 (403.5–788.0)	<0.001
Highest procalsitonin (ng/ml)	1.4 (0.22–7.84)	0.43 (0.13–1.63)	1.60 (0.33–8.50)	4.72 (0.61–16.6)	<0.001
Highest lactate (mmol/l)	3.2 (2.07–5.2)	2.50 (1.70–3.80)	2.4 (2.1–4.2)	4.25 (2.45–7.42)	<0.001
**Clinical outcomes**					
Mechanical ventilation, n (%)	98 (42.2%)	23 (29.1%)	27 (35.5%)	48 (62.3%)	<0.001
Death, n (%)	91 (39.2%)	20 (25.3%)	25 (32.9%)	46 (59.7%)	<0.001
Cardiogenic shock, n (%)	114 (49.1%)	23 (29.1%)	37 (48.7%)	54 (70.1%)	<0.001
Blood transfusion, n (%)	28 (12.1%)	9 (11.4%)	11 (14.5%)	8 (10.4%)	0.297
Dialyses, n (%)	34 (14.7%)	8 (10.1%)	6 (7.9%)	20 (26.0%)	0.529
Composite outcome, n (%)	124 (53.4%)	26 (32.9%)	41 (53.9%)	57 (74.0%)	<0.001
Total length of hospital stay, days	12.71 ± 10.37	9.60 ± 6.40	12.86 ± 8.17	16.40 ± 14.15	0.001
Length of stay in ward (days)	6.0 (2.0–8.0)	6.0 (3.0–8.0)	6.0 (3.25–9.0)	5.0 (0.0–8.0)	0.432
Length of stay in ICU (days)	3.0 (0.0–9.0)	0.0 (0.0–4.0)	3.5 (0.0–11.0)	7.0 (3.0–13.0)	<0.001

CAD: Coronary artery disease; CKD: Chronic kidney disease; COPD: Chronic obstructive pulmonary disease; CRP: C-reactive protein; eGFR: Estimated glomerular filtration rate; ICU: Intensive care unit; LDH: Lactate dehydrogenase; SBP: Systolic blood pressure; WBC: White blood cell.

**Figure 1.**
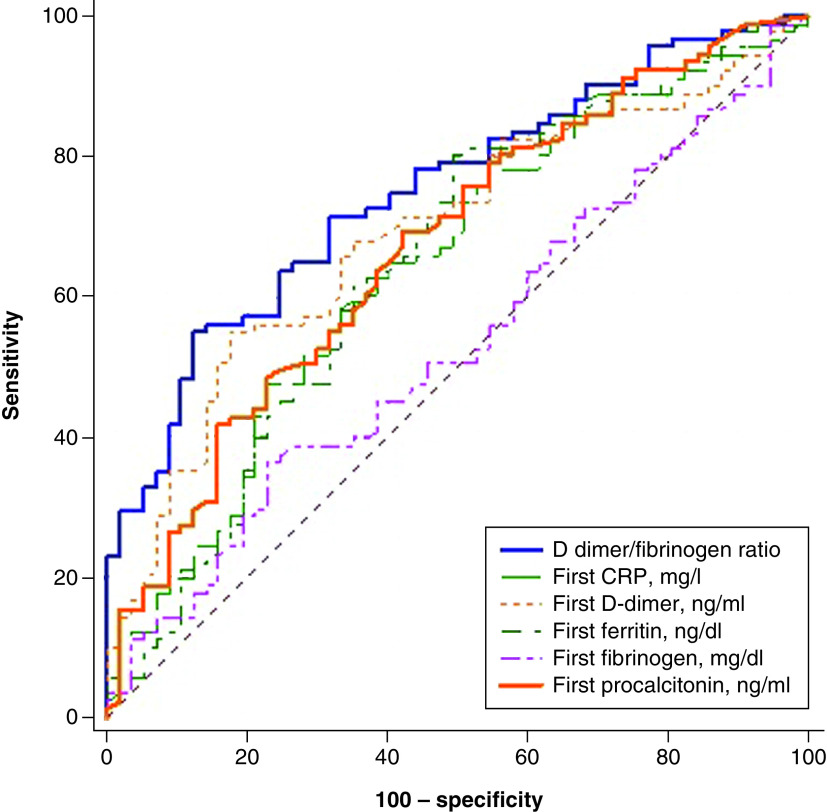
Receiver operating characteristic curve for significant markers in the prediction of clinical outcomes.

ROC curve analyses were performed to compare the predictive performances among DFR, D-dimer, fibrinogen and some traditional inflammatory markers, such as ferritin, CRP and procalcitonin (Figure 1). As presented in Table 2, cut-off values and the area under the ROC curve also were calculated. On the whole, DFR had a better predictive value than other markers.

**Table 2.** The cut-off values and area under the curve for D-dimer/fibrinogen ratio, D-dimer, fibrinogen, ferritin, C-reactive protein and procalcitonin.

Variables	cut-off values	AUC	Sen (%)	Spe (%)
D-dimer/fibrinogen ratio	0.61	0.741	47.6	90.7
D-dimer (ng/ml)	2.89	0.791	75.8	71.4
Fibrinogen (mg/dl)	546	0.682	62.4	73.4
Ferritin (ng/dl)	215	0.714	75.6	62
CRP (mg/l)	88.5	0.724	54.8	82.1
Procalcitonin (mg/ml)	0.202	0.704	70.5	61.9

AUC: Area under the curve; CRP: C-reactive protein.

**Figure 2.**
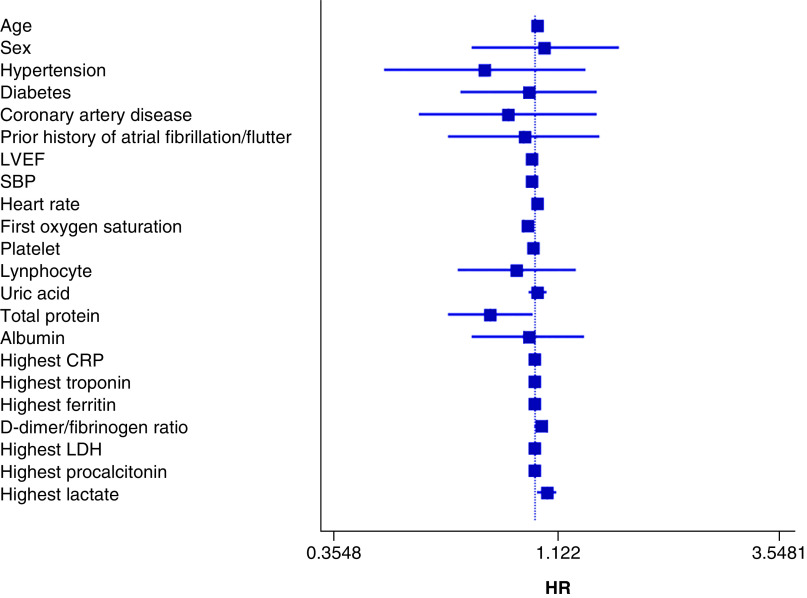
Forest plot of the univariate Cox proportional analyses for the composite of death, cardiogenic shock and intubation events in the total population (n = 232).

Based on cox proportional analyses; SBP (HR: 0.98; 95% CI: 0.97–0.99; p < 0.001), heart rate (HR: 1.01; 95% CI: 1.00–1.01; p = 0.013), admission oxygen saturation (HR: 0.96; 95% CI: 0.94–0.98; p < 0.001), highest procalcitonin (HR: 1.01; 95% CI: 1.00–1.03; p = 0.022), ferritin (HR: 1.00; 95% CI: 1.00–1.00; p = 0.001), lactate (HR: 1.06; 95% CI: 1.01–1.12; p = 0.020), CRP (HR: 1.00; 95% CI: 1.00–1.00; p = 0.045), total protein (HR: 0.79; 95% CI: 0.64–0.99; p = 0.041) and DFR (HR: 1.03; 95% CI: 1.00–1.07; p = 0.032) predicted clinical outcomes including death, cardiogenic shock and mechanical ventilation events in all populations (Figure 2). Multivariate cox analyses showed that the following were independent prognostic factors of outcomes: SBP (HR: 0.97; 95% CI: 0.96–0.99; p = 0.023), highest LDH (HR: 0.99; 95% CI: 0.99–1.00; p = 0.038) and DFR (HR: 1.07; 95% CI: 1.00–1.13; p = 0.027; Table 3).

**Table 3.** Enter methods of multivariate Cox proportional analyses for composite of death, cardiogenic shock and intubation events in the total population (n = 232).

Variable	p-value	Hazard ratio	95% CI	
			Lower	Upper
SBP, mmHg, mean ± SD	0.023	0.97	0.96	0.99
D-dimer/fibrinogen ratio	0.027	1.07	1.00	1.13
Highest LDH (u/l)	0.038	0.99	0.99	1.00

LDH: Lactate dehydrogenase; SBP: Systolic blood pressure.

## Discussion

**Figure 3.**
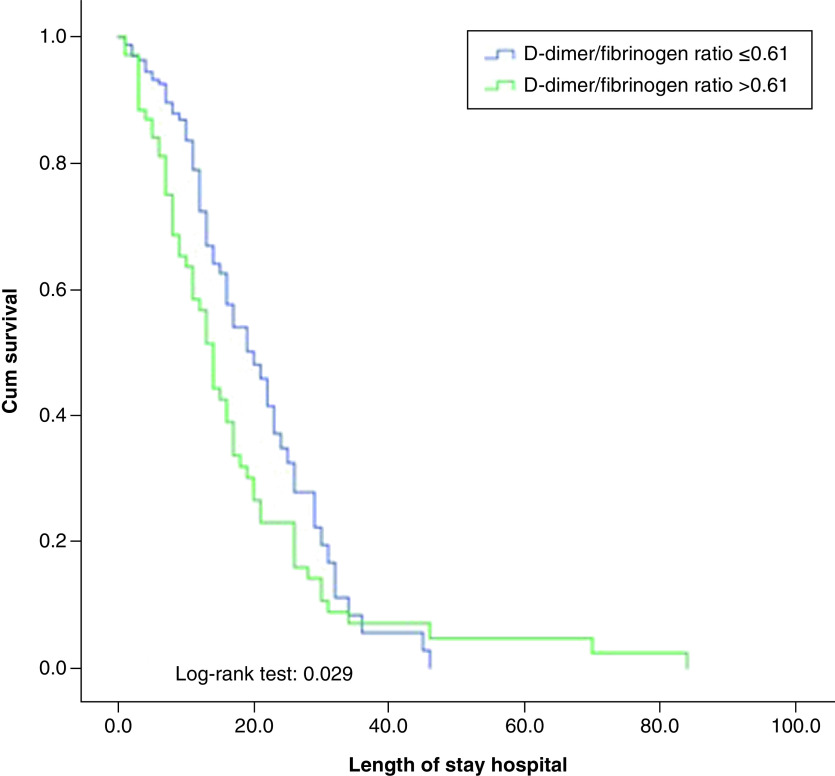
Kaplan-Meier survival curves for clinical outcomes in patients diagnosed with COVID-19 with a history of heart failure according to higher and lower D-dimer/fibrinogen ratio.

In this study, 27.5% of HF patients diagnosed with COVID-19 had a high DFR (>0.61) at admission and in patients with a history of HF diagnosed with COVID-19, DFR elevation upon admission was associated with poor clinical outcomes, longer hospital stays, length of stay in the ICU and in-hospital mortality (Figure 3). Previous studies have shown the correlation between plasma D-dimer and fibrinogen concentrations and the severity of COVID-19. Fibrinogen is an acute-phase protein, which is synthesized by the liver in response to IL-1- and IL-6-derived stimulation, and is involved in fibrin formation as the last step of a triggered coagulation activity [26]. Fibrinogen has become an important biomarker in the course of COVID-19 disease, as it is associated with both inflammation and coagulopathy. Han *et al.* investigated the changes in blood coagulation of patients infected with COVID-19 by comparing them with healthy controls. They reported that fibrinogen levels were higher in both mild and severely ill patients than healthy patients [17]. In another study, the difference in fibrinogen was reported to be nonsignificant between surviving and nonsurviving patients with COVID-19 in a different cohort (5.16 vs 4.51 g/l, p = 0.149) [18]. Hayıroglu *et al.* reported that fibrinogen might not have a predictive value for mortality in patients with COVID-19 and should be evaluated together with D-dimer for proper prognostic assumption [19]. Although the mortality rate in the third tertile DFR group was 59.7%, there was no difference in fibrinogen value between the groups. In addition, fibrinogen alone was not found to be predictive in terms of mortality and clinical outcomes.

D-dimer, which is produced by the breakdown of fibrin by plasmin, is another biomarker closely related to thrombotic and fibrinolytic processes. Apart from the diagnosis of coagulation abnormalities, D-dimer increases in many conditions such as inflammation, vasculitis, pregnancy, cancer and HF. Several studies have shown that D-dimer levels are associated with the severity and clinical outcomes of community-acquired pneumonia [27]. Among adults admitted to the emergency room, infection,s rather than venous thromboembolism (VTE)/pulmonary embolism (PE), are the most common reason for D-dimer elevation [28]. In addition, some studies have reported that a high D-dimer level is a highly nonspecific marker of VTE and may be a sign of inflammation rather than thrombosis [29,30]. Many studies have previously evaluated the relationship between COVID-19 and D-dimer. In some studies, the level of D-dimer reflects the severity of the disease, while in others it is useful in predicting in-hospital prognosis. A large study of 1065 hospitalized patients with COVID-19 reported that higher D-dimer at admission was associated with a greater risk of all-cause mortality, need for mechanical ventilation and VTE. The investigators also concluded that D-dimer at admission, as an isolated measure, did not appear to be a reliable prognostic test for outcomes among patients with COVID-19 [31]. Although D-dimer has been well studied, the predictive effect of DFR in COVID-19 disease has not been examined. Furthermore, despite the results of these studies, the prognostic and predictive value of D-dimer has not been studied in patients with COVID-19 and specific diseases. To the best of our knowledge, this study is the first to investigate the association of DFR with the outcomes of patients with HF and COVID-19.

In this study, the AUC was found to be 0.74 for the ROC of DFR at admission, which is considered a predictor of in-hospital mortality. The optimal DFR cut-off value of 0.61 provided sensitivity and specificity for the prediction of in-hospital outcomes. Unlike previous studies in which D-dimer was evaluated, this study was conducted in a specific patient population with high in-hospital poor outcomes. A study of 343 inpatients with COVID-19 from Wuhan reported that AUC of 0.89 for a ROC of D-dimer at admission as a predictor of in-hospital mortality. They also reported that the optimal D-dimer cutoff of 2 μg/ml for prediction of death. However, these findings were based on only 13 death events in the cohort – hardly sufficient to construct a rigorous and reliable ROC [32]. In another study of 138 consecutive patients with COVID-19 indicated that ‘D-dimer was higher in non-survivors than in survivors’, however, this was based on a subgroup analysis that included 33 patients, only five of whom were nonsurvivors [33]. In the present study of patients with HF, (which is an important group in terms of COVID-19 mortality), high DFR at admission was more specific than D-dimer and fibrinogen in predicting clinical outcomes, such as requiring mechanical ventilation, cardiogenic shock, in-hospital death, and length of hospital and ICU stay.

Severe COVID-19 is commonly complicated with coagulopathy, but the elevation of D-dimer seen in patients with COVID-19 may be associated with inflammation rather than venous thromboembolism [33,34]. In a study that included 449 patients with COVID-19, there was no difference in D-dimer at admission as compared with 104 patients with non-COVID pneumonia [34]. Likewise, the last two studies showed that D-dimer correlates with inflammatory markers, such as CRP and procalcitonin, and there is no definitive conclusion about its direct relationship with VTE [31,35]. In the present study, a high DFR value was significantly correlated with prognosis-determinant biomarkers of both inflammation and disease, such as CRP, LDH, procalcitonin, lactate, troponin and ferritin. When evaluated together with these parameters, DFR was found to be associated with in-hospital outcomes in both univariate and multivariate analyses. Inflammatory activation may be the underlying cause of worse clinical outcomes in both patients with HF and those with COVID-19.

## Limitations

The present study has several limitations. First, it is a retrospective, observational study. Second, there was a lack of evaluation of conditions such as in-hospital undiagnosed PE and VTE. This may have contributed to the dynamic changes in fibrinogen and D-dimer levels observed in this study. Further studies are needed to confirm these findings.

## Conclusion

The DFR value at admission may guide physicians in determining the outcomes, length of hospital stay, treatment protocol and regimen for patients with a history of HF who are admitted with a diagnosis of COVID-19.

**Summary points**

- Heart failure is one of the most important causes of mortality in patients with COVID-19.
- Previous studies have shown that fibrinogen and D-dimer are associated with the severity of COVID-19 disease; however, the prognostic value of DFR in patients with COVID-19 with a history of heart failure is unclear.
- Elevated DFR values may define prothrombotic activity in conditions with excessive fibrinogen consumption and D-dimer formation, independent of the absolute values of both.
- Laboratory parameters of patients with COVID-19 have shown a prothrombotic diathesis with significantly high fibrinogen levels in critically ill patients. However, in the late stages, thrombolysis decreases fibrinogen levels and increases D-dimer.
- Multivariate Cox regression analysis showed that DFR was significantly associated with in-hospital outcomes.
- DFR can be used as a biomarker in patients with COVID-19 and heart failure, which is associated with increased mortality and worse outcomes.
